# "It's not even about her it's about the whole family": accounts of participation in a family cancer study

**DOI:** 10.1186/1897-4287-10-S2-A14

**Published:** 2012-04-12

**Authors:** H Shipman, A Clarke

**Affiliations:** 1 School of Medicine, Cardiff University, Wales, UK

## 

Accounts of participation in genetic research are dynamic and innately social. Participation is viewed not as an individualised act but as morally significant and involving relational responsibilities. Previous work has investigated individuals’ motivations for research participation in terms of such relational complexities. Here we explore the significance of relational responsibility in the context of participation in a familial cancer study where many family members are eligible for recruitment. We consider accounts of family (non)participation, (im)morality and (ir)responsibility. By looking at accounts we can explore the ways in which people use explanations to construct social reality. Accounts are more than disclosing internal attitudes, rather they provide claims of moral worth and participation in the social world. The accounts are taken from eight in depth interviews with participants from the kConFab Study, investigating familial breast cancer.

Interviewees gave the juxtaposed accounts described elsewhere, with motivations for research participation framed in terms of self, family and society. In addition to accounts relating to themselves, all interviewees accounted for family members’ (non) participation. Accounts of self and relatives’ participation in the research were brief, with strongly altruistic framing and participation described as meeting expectations. Four participants knew of family members who had chosen not to participate. Accounts about this were longer and involved more elaborate moral character work. As research participation was framed as beneficial to the family and others, with minimal burden to the participant, excusing and justifying non-participation was required in order to explain it. This was done through descriptions of illness, disability, relatives’ feelings of guilt and logistics. In one instance a sister’s non-participation was not justified or excused but explained it in terms of her ‘selfishness’. Reports of non-participation by relatives involved negative feelings towards them, ranging from frustration to anger. Stronger emotional responses were described when the relative was perceived as having particular responsibilities towards the interviewee or their offspring, as a parent or sibling.

The familial tensions described in the accounts are part of a broader narrative of pre-existing difficulties in family dynamics. The genetic research context became another field in which these difficulties became evident or entrenched. Our findings inform our understanding of how genetic research may be experienced within a family. With participation framed as acting responsibly towards others there may be relational ramifications if individuals decline to do so. This has implications for how consent is viewed and how studies may choose to recruit participants.

